# Exploring the effectiveness of a fitness‐app prototype for home care service users in Austria and Italy

**DOI:** 10.1111/hsc.13733

**Published:** 2022-01-30

**Authors:** Birgit Trukeschitz, Siegfried Eisenberg, Cornelia Schneider, Ulrike Schneider

**Affiliations:** ^1^ Research Institute for Economics of Aging WU Vienna University of Economics and Business Vienna Austria; ^2^ Institute of Computer Science University of Applied Sciences Wiener Neustadt Wiener Neustadt Austria; ^3^ Institute for Social Policy and Research Institute for Economics of Aging WU Vienna University of Economics and Business Vienna Austria

**Keywords:** Active and Assisted living (AAL), digital behavioural change intervention (DBCI), health behaviours, home care, physical activity, randomised controlled trial (RCT), technology

## Abstract

An infinite number of fitness apps are available on various app stores. However, hardly any of them are fitted to the needs and requirements of care‐dependent people. This paper investigates the effectiveness of a customised fitness‐app prototype for increasing physical activity in home care service users. Home care service users from Austria and Italy were randomly assigned to two groups. In total, 216 participants were involved in the field trial, 104 received a tablet with the fitness app and an activity tracker (treatment group), 112 did not (control group). Regularity of physical activity, frequency of fitness exercises and walking behaviour were self‐reported by participants at baseline, after 4 months and after 8 months. In addition, the frequency of using the prototype was assessed based on the fitness app's logged usage data. We estimated multilevel mixed‐effects ordered logistic models to examine the effects of the intervention. After 4 months, the intervention increased the home care users’ probability of agreeing strongly with being physically active on a regular basis by 28 percentage points (*p* < 0.001; 95% CI: 0.20, 0.36) and their probability of reporting to exercise more than once a week by 45 percentage points (*p* < 0.001; 95% CI: 0.32, 0.57). Walking behaviour was not affected on group‐level but improved for frequent users of the activity tracker. Frequent and regular users of the fitness app benefited most and effects persisted until the end of the 8 months controlled trial. Tailoring a fitness‐app prototype to the needs of care‐dependent people has the potential to support people with functional limitations to engage in a more active lifestyle. Future research is encouraged to seek further insights into how new technologies can support physical activities in people with long‐term care needs.

**What is known about this topic**

- Digitisation shapes our lives in many areas, however, less profoundly so far in social care settings.
- Physical activity is known to be key for a healthy life, also for older people's cognitive and physical functioning and people with chronic conditions and care needs.
- Traditional face‐to‐face exercise programs face challenges when delivered to community‐dwelling care‐dependent people. Supportive effects of new technologies promoting exercising have been shown for younger groups of older people.

**What this paper adds**

- The effectiveness of a fitness‐app prototype is explored in home care service users – a group that has vastly escaped the attention of previous digital interventions research.
- Fitness apps have the potential to increase physical activity for older care‐dependent people living in the community if developed to the needs and abilities of this group.
- Combining self‐assessed levels of physical activity with objectively measured usage of digital fitness systems allowed to gain detailed insights into drivers of the effects.

## 1 INTRODUCTION

Population ageing implies that the number of people in need of long‐term care (LTC) will further increase in the years ahead (OECD, 2017). This calls for prevention, policy innovation, and a new range of solutions for LTC provision (Bloom et al., 2015; Trencher & Karvonen, 2018). One approach, most prominently denoted as eHealth, focuses on the role of information and communication technologies (ICT) in improving health as well as healthcare processes and the value of health for societies (European Commission, 2021).

‘Active and Assisted Living’ (AAL, former Ambient Assisted Living) technologies, particularly aim to support ageing in place by aiming to postpone frailty and cognitive decline and to improve quality of life (Siegel & Dorner, 2017) by tailoring ICT‐solutions to the requirements of older people. Predominately, AAL‐technologies seek to enhance comfort or support, but some are geared up to maintain or improve older people's competencies and abilities by encouraging activity (Leitner et al., 2015). Particularly, physical activity is known to be key for a healthy life due to its broad range of benefits for older people's cognitive and physical functioning (Bherer et al., 2013; Langlois et al., 2012; Marsillas et al., 2017), and for people with chronic conditions and care needs (Cederbom et al., 2017; de Souto Barreto et al., 2016).

Traditional face‐to‐face exercise programmes for daily use reach their limits (Stockwell et al., 2019), especially when delivered to community‐dwelling care‐dependent people. Take‐up is affected by limitations to get around, which restrict going to other places, and the cost‐intensiveness of home visits. Thus, strategies are needed to make it easier for community‐dwelling care‐dependent people to benefit from exercising and other physical activities.

A systematic review of digital behavioural change techniques for promoting physical activity concluded that such interventions have attracted specific attention as a potentially scalable and low‐cost alternative to traditional face‐to‐face approaches (Stockwell et al., 2019). Evidence also highlights positive effects of prototyped internet‐based interventions (Broekhuizen et al., 2016; Hansen et al., 2012; Kwan et al., 2020; Maher et al., 2015) and mobile health applications (‘mHealth’ apps, as part of eHealth approaches; Elavsky et al., 2019; Hong et al., 2015; King et al., 2016; Kwan et al., 2020; Sullivan & Lachman, 2017) on physical activity behaviour of older adults.

However, while there is an almost infinite number of fitness apps available on various app stores, hardly any of them are fitted to the needs and requirements of care‐dependent people (Klimova, 2018). Likewise, previous digital behavioural change trials mainly addressed people in their early 60s not needing help or support. According to Kwan et al.’s (2020) systematic review of 38 randomised controlled trials (RCTs), two out of three trials focused on healthy older people. Against this backdrop, Elavsky et al. (2019) call for mobile health interventions targeting underserved groups of older adults.

This paper explores as one of the first whether a fitness‐app prototype tailored to the requirements of care‐dependent people increases self‐assessed levels of physical activity in home care service users. More specifically, it investigates the effects of the fitness‐app prototype on users’ perceptions of their (i) overall level of physical activity, (ii) frequency of exercising at home and (iii) frequency of walks. Furthermore, it uses data from the app's log to capture the usage of both the digital exercise program and the activity tracker. This is in line with a recommendation to include usage metrics in eHealth trials (Eysenbach, 2005). Thus, this paper is one of very few paying particular attention to the frequency of using a fitness app and its impact on the assessment of outcomes in care‐dependent people. While previous evidence is restricted to mainly short‐term trials, lasting from two to 3 months (Broekhuizen et al., 2016; Maher et al., 2015), our trial period spanned 8 months to also investigate the persistence of effects.

## 2 CONCEPTUAL UNDERPINNING OF THE INTERVENTION AND HYPOTHESES

In this paper, behaviour change theory is used to better understand whether an ICT‐supported fitness program has the potential to influence behaviour. Interventions rooted in behaviour change theory are expected to be more effective than interventions without this conceptual underpinning (de Korte et al., 2018). Studies investigating selected features related to behavioural change techniques (BCTs), such as activity overviews and recommendations for beneficial activity levels, reported increased levels of physical activity of care‐dependent people (de Korte et al., 2018; Sullivan & Lachman, 2017). Interventions applying several BCTs led to larger effect sizes (Webb et al., 2010). Findings from systematic reviews and meta‐analyses also support the use of multiple BCTs when designing digital solutions to increase physical activity in older adults (Muellmann et al., 2018; Stockwell et al., 2019; Sullivan & Lachman, 2017) and suggest to diversify the set of BCTs (Zaslavsky et al., 2020).

Accordingly, the fitness‐app prototype investigated in this paper was expected to increase physical activity in care‐dependent people through a set of BCTs grounded in health behaviour change theory. We assigned the features of the fitness app (implemented on a tablet and supplemented with an activity tracker) to the BCTs taken from Michie et al.’s (2011) taxonomy and grouped them into five clusters: information‐related, activity‐related, incentive‐related, BCTs affecting the self and social‐related BCTs.

*Information‐related BCTs* aim to shape the users’ knowledge of physical activity in general and its positive consequences. To this end, the fitness‐app prototype provided tips of the week and (via the tablet's screensaver) 10 supporting facts for being active. The app further included visual and audio instructions on how to perform each exercise. Furthermore, it provided feedback and monitoring features, such as the number of completed exercises after each training session. In addition, a summary activity overview showed the time spent on using the exercise programme, activities, and steps, each on a daily, weekly and monthly basis. A fitness tracker paired with the fitness app offered a customised watch face to facilitate monitoring activities. The fitness system enabled participants to automatically or manually track standard activities, such as walking.

*Activity‐related BCTs* referred to the daily alternating exercise program to improve balance, strength and coordination skills, reflecting the skill level of the users (Jungreitmayr et al., 2021). The exercise programme and tips of the week aimed to provide attractive alternatives to sedentary behaviour.

*Incentive‐related BCTs* comprised rewards, particularly bronze, silver and golden trophies available on the app's summary activity overview and its start screen.

*BCTs affecting the self* were implemented to support older people to cope with technology and exercise, by using four approaches. First, to facilitate access, the app was installed with a launcher that replaced the home screen of the tablet with the app's home screen. Second, a fitness coach selected exercises and the number of repetitions to match users’ fitness levels. Third, videos showed instructors of approximately the same age or a few years younger to strengthen users’ confidence in being able to do the exercises and thus create a valued self‐identity. Finally, automated encouragement complemented the numeric feedback on performed exercises, motivating the user to keep going or to increase physical activity.

*Social‐related BCTs* played only a minor role, as the fitness app was designed for training sessions to be completed by users on their own, with no online comparisons with other users. Only home care service users’ regular care workers introduced them to the fitness app over a period of six weeks.

The fitness app features were created to support a more active lifestyle in care‐dependent people by encouraging indoor and outdoor activities. As activities like exercises and walks that last longer than 10 min positively affect health (WHO, 2010), this threshold was considered by the app's design. Thus, if the fitness‐app prototype worked as planned, care‐dependent users should be able to increase their physical activity levels.

For evaluating the effectiveness of this digital behavioural change intervention (DBCI), we selected three outcome indicators: (i) regularity of physical activity, (ii) frequency of doing exercises and (iii) frequency of walks lasting 10 min or longer. We expected users of the fitness app to more likely report *engaging in physical activities on a regular basis* (hypothesis 1a). In addition, we expected *frequent and regular users* of the summary overview of physical activities to more likely report *regular physical activity* than non‐users (hypothesis 1b). Second, we hypothesised that the fitness app, particularly its exercise program, the summary overview of completed exercises, and the rewards, would contribute to a *higher* self‐assessed *frequency of practicing fitness exercises* (hypothesis 2a), especially for *frequent and regular users* of the fitness exercise function (hypothesis 2b). Thirdly and finally, we hypothesised that the fitness app would *increase the frequency of walks lasting longer than 10* *min* (hypothesis 3a), specifically for people using the *wearable activity tracker frequently or at least regularly* (hypothesis 3b).

## 3 METHODS

### 3.1 Study design

The experimental study was conducted with 216 home care service users in Salzburg (Austria) and Lombardy (Italy) in 2017/18. The treatment group (TG) received the fitness‐app prototype, including a paired activity tracker. The control group (CG) had no access to the fitness technology. The University of Salzburg's ethics committee (EK‐GZ 30/2016) approved the study design.

We targeted home care service users between 55 and 85 years who may be limited in mobility, i.e., they may use walking and mobility aids except for wheelchairs. To assure that older people could participate in the field trial, participants had to have at most low visual/hearing deficiencies and no cognitive impairments (Trukeschitz & Blüher, 2018a).

Eligible home care service users were randomly assigned to the trial or control arms, using SPSS 24.0 (IBM, 2016). As a first approximation, a priori power analysis for *t*‐tests using G*Power (Faul et al., 2009) for medium effect sizes (*d* = 0.05) with an alpha of 0.05 called for 88 participants per group. To allow for drop‐out, we planned to involve as many home care service users as possible. As our study addressed vulnerable people, participation in general could present a burden. Given the potential cognitive and physical challenges of the intervention, we expected a higher rate of decline in the TG. To deal with such assignment non‐compliance, we used matching procedures prior to recruitment to identify statistical twins from the CG and people allocated to the TG. Care workers were instructed to recruit the participants according to the recruitment list. In the event that people in the TG did not agree to participate, their statistical twins could be approached as potential substitutes.

Data for the effectiveness analysis were collected via paper‐and‐pencil surveys at three time points: before the field trial started, 4 months into the controlled trial, and after 8 months at the end of the intervention. Questionnaires were administered by the home care service users’ care worker team (Trukeschitz & Blüher, 2018a).

### 3.2 Intervention

TG participants received the fitness‐app prototype on a tablet (Samsung Tab A 2016) and a customised activity tracker (Samsung Gear Fit2), paired with the fitness app, for 8 months. The fitness app prototype comprised video clips of fitness exercises, a summary overview of physical activities and rewards, and a weekly tip to promote physical activity in general (Schneider et al., 2020; Trukeschitz & Blüher, 2018a). The exercise programme consisted of six daily changing exercises with two repetitions each. To move on to the next exercise, users either had to confirm the completion of the exercise (for exercises that required a certain number of repetitions), or start a countdown (e.g. 40 s), or skip the exercise. The program included a built‐in hidden time count, which ensured that completed exercises were only counted after a pre‐set reasonable time for doing this exercise (on average, 30 s). The app also provided access to electronic newspapers, the internet and games (Schneider et al., 2020). The CG received no technology, but shopping vouchers after completing all three questionnaires.

Care workers introduced TG participants to the fitness app at their homes and explained selected features once a week over a period of six weeks. From then on, personal contact related to the prototyped app was held to a minimum to reduce potential influence other than the technology. In addition, no other people were involved in explaining the app other than those visiting the household as part of their care work routine.

### 3.3 Measures

**FIGURE 1:**
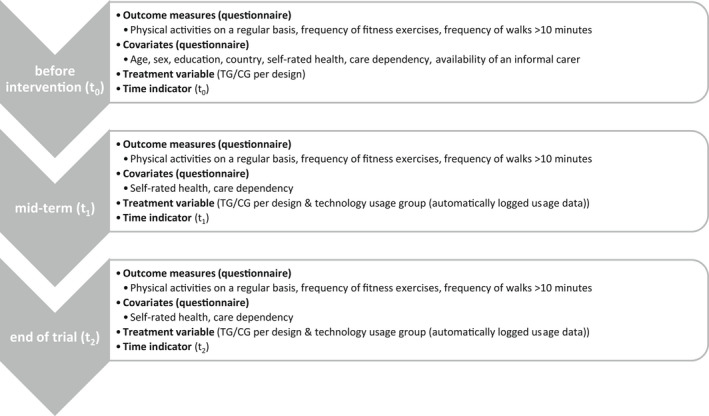
Overview of variables collected at each time point

Figure 1 gives an overview of the measures used in the models which are described in detail below.

### 3.4 Outcome measures

We collected data on three self‐assessed outcomes. First, participants in TG and CG rated the *regularity of their physical activity in general* on a five‐point Likert scale by indicating their level of agreement with the statement ‘I am regularly physically active to stay healthy as long as possible’ (‘strongly disagree’ (0) to ‘strongly agree’ (4)). Second, participants were asked to indicate their *actual frequency of fitness exercises* in the past four weeks on a scale with four response options (from ‘never’ (0) to ‘more than once a week’ (3)). Finally, to cover the level of outdoor activity, participants were asked to rate their *walking behaviour for walks lasting at least 10* *min* on a five‐point scale (from ‘less than several times a month’ (0) to ‘daily’ (4)).

### 3.5 Treatment and time indicators

We measured two effects on each of the three outcomes for all participants who finished the controlled trial: the treatment effect of the fitness‐app prototype and the effect resulting from the frequency of using the fitness app. For assessing the *group‐specific treatment effect*, we included an indicator for the TG. For assessing the effect resulting from the *frequency of using* the fitness programme (Schneider et al., 2020), we employed logged usage data of both the activity tracker and two features of the app that closely relate to the outcome indicators (‘summary overview of physical activities’ and ‘fitness exercises’). We identified four user groups on a monthly basis: ‘frequent’ (3), ‘regular’ (2), ‘infrequent’ (1), ‘non’‐users (0), for each app feature and the activity tracker (Schneider et al., 2020). Based on WHO (2010) recommendations, a participant is qualified as a frequent, regular or infrequent user of the exercise function when using the feature ‘at least 8 times’, ‘4 to 7 times’ or ‘1 to 3 times’ per month, respectively. For the summary overview of physical activities and for the activity tracker, the according usage intervals were ‘at least 20 times’, ‘10 to 19 times’ and ‘1 to 9 times’ per month. Participants in the CG were allocated to the non‐user group. An additional variable indicated the time of data collection (before (*t*_0_), mid‐term (*t*_1_) and at the end of trial (*t*_2_)).

### 3.6 Covariates

Covariates were chosen to capture the influence of age, sex, education, health and care dependency on physical activity (Biernat & Tomaszewski, 2011; Franco et al., 2015; Leis et al., 2010; McKee et al., 2015) and on technology use (Elliot et al., 2014; Kavandi & Jaana, 2020; Kim et al., 2017). A *country dummy* (‘0’ for Austria, ‘1’ for Italy) accounted for organisational, institutional and structural differences.

As to the sociodemographic variables, *age* was calculated and treated as constant over the trial period of 8 months, so were *sex* (coded ‘0’ for men and ‘1’ for women) and *education level* (based on the International Standard Classification of Education 2011; ISCED, 2011). *Self‐rated health*, measured by the six‐level general health assessment item of the SF‐36 questionnaire (Ware & Sherbourne, 1992), was adjusted for changes over time. In line with Hershey et al. (2010), we treated self‐rated health as a metric variable to model the relationship between health and the outcome variables.

A *care dependency index* draws on eight items reflecting abilities to cope with (instrumental) activities of daily living, (I)ADLs. The (I)ADL items captured self‐rated abilities for getting up, taking a shower, getting dressed, walking up/down stairs, shopping, house cleaning and handling finances. Each (I)ADL item had four response options. We formed an additive index and calculated the average for each participant at the baseline, after 4 and 8 months (‘0’ refers to ‘completely dependent’ and ‘3’ to ‘completely independent’). A dummy accounted for the *availability of an informal carer*.

### 3.7 Statistical analyses

The number of missing items did not exceed 3% for any outcome level at any time. Little's MCAR‐Test (Little, 1988) confirmed that all missing items could be treated completely at random. We performed Wilcoxon rank‐sum tests and chi‐square tests to investigate potential differences between groups at baseline.

For the effectiveness analyses, we estimated mixed‐effects ordered logistic models with clustered, robust standard errors (Gelman & Hill, 2006). Within our multilevel (ML) models, we considered repeated observations as nested within individuals. Furthermore, covariates were added to adjust for confounding effects.

We fitted two types of ML‐models. *ML‐model 1* assessed the *treatment effect* of the intervention between groups (TG/CG) using a differences‐in‐differences equation structure (Angrist & Pischke, 2008). The model included a dummy indicator for the TG, the time indicator, the interaction of group and period, and previously described covariates to consider potential selection effects (Angrist & Pischke, 2008; Mood, 2010; Wooldridge, 2002). A random intercept was specified at the individual level. We estimated the effects for the first 4 months (*t*_0_ − *t*_1_) and for the whole trial period of 8 months (*t*_0_ − *t*_2_). We further explored whether the effect resulted from a change in outcomes in the TG only or, additionally, from a change in the outcomes of the CG over time. For more detailed insight into the effect, we predicted the probabilities for each outcome level for each group.

*ML‐model 2* assessed the *effect of the frequency of using* the fitness app. The estimation included the usage groups (non‐, infrequent, regular and frequent users), with non‐users as the reference group, the time indicator and all covariates of ML‐model 1.

For both ML‐models, we calculated average marginal effects to examine the treatment effect. Hence, we reported on the change in probabilities between TG and CG for certain outcome levels (ML‐model 1) and the change in probabilities comparing frequent‐, regular‐, infrequent‐users to non‐users (ML‐model 2). As some of the outcome categories had low numbers of observations, we tested for robustness by also running the models with collapsed response categories. As the results did not change, we kept the models with the original outcome categories. All statistical analyses were performed using Stata 15 (StataCorp, 2017); a *p*‐value of less than 0.05 was considered statistically significant.

## 4 RESULTS

**FIGURE 2:**
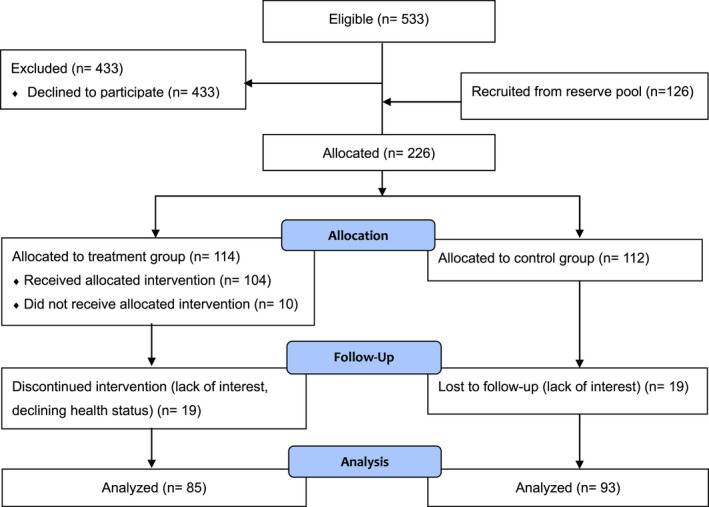
Participant flow chart

Figure 2 shows the participant flow. In total, 533 users of home care services met the eligibility criteria and were randomly assigned to TG and CG prior to recruitment. As recruitment reached its limit, given the target to involve at least 176 participants, we invited additional home care service users (‘reserve pool’), slightly relaxing the eligibility criteria and using a matching procedure. In total, 226 people agreed to be involved in the 8‐month controlled trial. Of those, 212 started (TG: 104 and CG: 112), and of those 178 people (TG: 85 and CG: 93) completed the trial after 8 months.

### 4.1 Sample descriptive statistics

Table 1 shows the sample descriptive statistics at baseline, with similar characteristics in TG and CG for sociodemographic variables, health, and also for the dependency score. Participants in the TG, however, were significantly more likely to have an informal carer (*p* < 0.001).

**TABLE 1:** Baseline characteristics of the participants of the TG and CG

	TG *n* = 85	CG *n* = 93	*p*‐value
*Demographics*			
Age (years), mean (*SD*)	74.3 (7.7)	75.4 (7.3)	0.358
Max.	91	88	
Min.	49	56	
Sex, %			0.304
Female	74.1	65.6	
Male	25.9	34.4	
Education, % (ISCED)			0.200
Lower secondary (0–2)	36.6	37.7	
Upper secondary (3)	31.2	24.7	
Post secondary (4)	14	25.9	
Tertiary (5–8)	14	9.4	
Missing	4.3	2.4	
Country, %			0.780
Austria	63.5	61.3	
Italy	36.5	38.7	
*Health*			
Health, mean (*SD*)	3.3 (0.9)	3.2 (0.9)	0.409
Max.	6	6	
Min.	2	1	
*Dependency score*			
(I)ADL‐Score, mean (*SD*)	2.3 (0.7)	2.2 (0.6)	0.306
Max.	3	3	
Min.	0.4	0.4	
*Informal support*			0.001
Informal Carer, %			
Yes	82.4	58.1	
No	15.3	40.9	
Missing	2.4	1.1	
*Outcomes*			
Physical activities on a regular basis, % (*n*)			0.295
Strongly agree	14.1 (12)	22.6 (21)	
Agree	24.7 (21)	16.1 (15)	
Somewhat agree	14.1 (12)	20.4 (19)	
Disagree	20.0 (17)	19.4 (18)	
Strongly disagree	23.5 (20)	18.3 (17)	
Missing	3.6 (3)	3.2 (3)	
Frequency of fitness exercises, % (*n*)			0.195
More than once a week	27.1 (23)	17.2 (16)	
Once a week	4.7 (4)	11.8 (11)	
Less than once a week	27.1 (23)	30.1 (28)	
Never	36.5 (31)	37.6 (35)	
Missing	4.7 (4)	3.2 (3)	
Frequency of walks longer than 10 min, % (*n*)			0.509
Daily	23.5 (20)	31.2 (29)	
Several times a week	37.7 (32)	28.0 (26)	
Once a week	11.8 (10)	19.4 (18)	
Several times a month	8.2 (7)	7.5 (7)	
Less than several times a month	12.9 (11)	11.8 (11)	
Missing	5.9 (5)	2.2 (2)	

Note

Percentages may not total 100 due to rounding.

On average, participants in both groups were about 75 years old, some 70% were women, and more than 60% were from Austria. Almost all participants met the target group criteria. Only one participant in the TG was younger than 55 years old and four were older than 85 years old in each group.

At baseline, TG and CG did not significantly differ for any outcome indicator. As Table 1 shows, both groups reported low initial levels of exercise and physical activity. About a third of each group reported going for a 10‐min or longer walk once a week or even less often. Thus, there was substantial potential for activation.

Table 2 displays the distribution of ‘frequent’, ‘regular’, ‘infrequent’ and ‘non‐’ users per feature. After 4 and 8 months, more than half of the TG were ‘frequent’ or ‘regular’ users’ of these fitness app features and the activity tracker. Usage decreased slightly between *t*_1_ and *t*_2_, more noticeably for the activity tracker and the summary overview of physical activities.

**TABLE 2:** Fitness app user groups (*t*_1_ and *t*_2_), TG only (*n* = 85)

Fitness‐app prototype feature	User type	After 4 months (*t*_1_)	After 8 months (*t*_2_)	change *t*_1_/*t*_2_ *p*‐value
		% (*n*)	% (*n*)	
Summary overview of physical activities				<0.001
	Frequent	54.1 (46)	43.5 (37)	
	Regular	24.7 (21)	24.7 (21)	
	Infrequent	14.1 (12)	16.5 (14)	
	Non‐user	7.1 (6)	15.3 (13)	
Fitness exercises				<0.001
	Frequent	56.5 (48)	52.9 (45)	
	Regular	15.3 (13)	8.2 (7)	
	Infrequent	12.9 (11)	12.9 (11)	
	Non‐user	15.3 (13)	25.9 (22)	
Activity tracker				<0.001
	Frequent	47.1 (40)	37.7 (32)	
	Regular	23.5 (20)	17.7 (15)	
	Infrequent	20.0 (17)	18.8 (16)	
	Non‐user	9.4 (8)	25.9 (22)	

Note

Percentages may not total 100 due to rounding.

### 4.2 Model results

**FIGURE 3:**
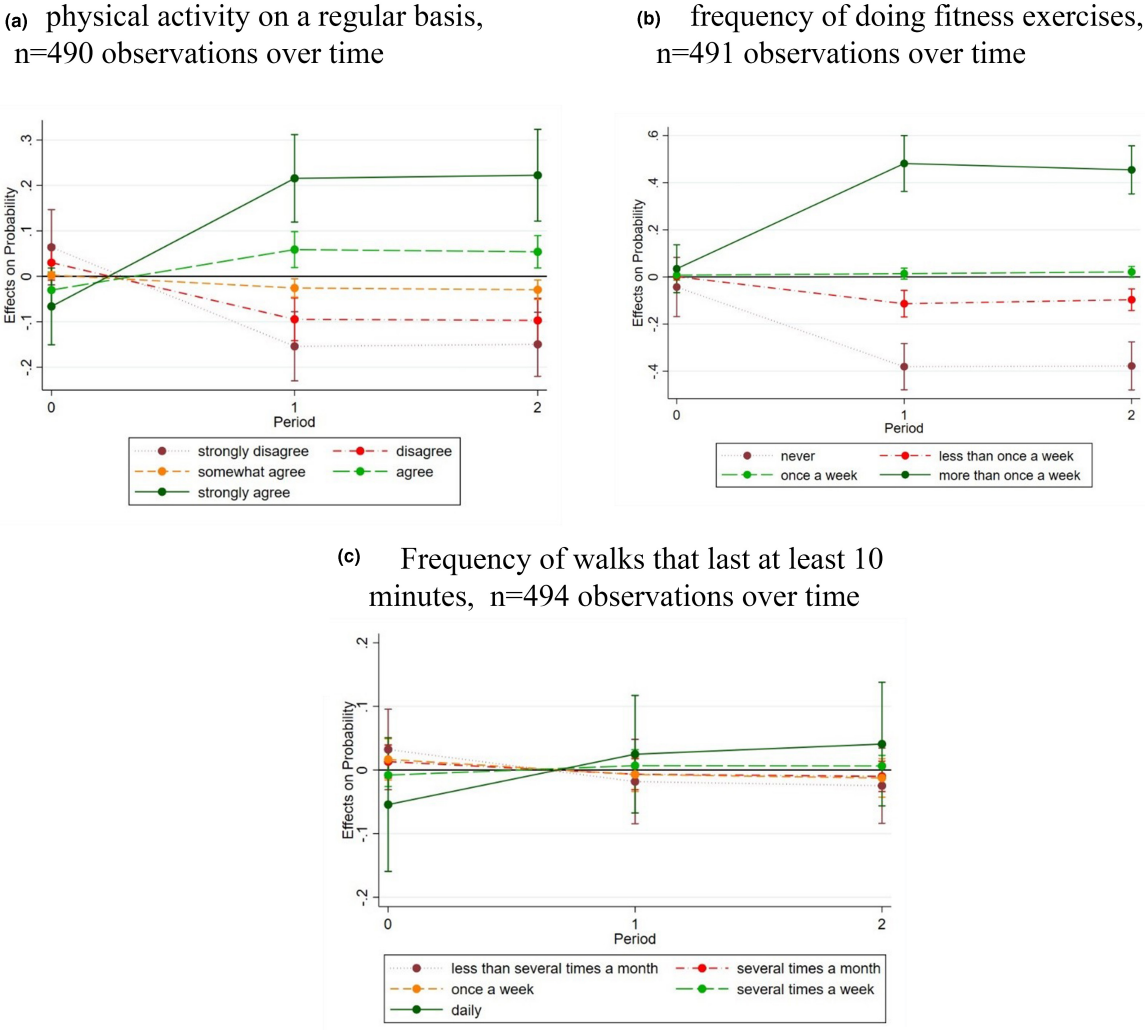
Differences in the predicted probability for each outcome level between TG and CG over time, model type 1. Source: WU, CiM effectiveness surveys data. (a) physical activity on a regular basis, n = 490 observations over time. (b) frequency of doing fitness exercises, *n* = 491 observations over time. (c) Frequency of walks that last at least 10 min, *n* = 494 observations over time. Note: responses of the TG contrasted with responses of the CG which are represented by the zero‐line

In presenting the estimated treatment effects, we refer to average marginal effects, that is, changes in probabilities over time, for regular engagement in three types of physical activity between TG and CG. Figure 3a–c depicts the results for ML‐model 1 (not accounting for frequency of use) in a clear and succinct form. The zero‐line in Figure 3 represents the CG values. We provide the estimated changes in probabilities in percentage points and the corresponding p‐value and confidence interval (95% CI). Tables S1 and S2 in the Supporting Information section contains the results of the underlying multi‐level ordered logistic regression (ML‐model 1 and ML‐model 2).

### 4.3 Effects on regular physical activity

At the beginning of the trial, the probability of being active on a regular basis was reported to be about the same for the TG and CG (Table 1, and Figure 3a). After 4 months (*t*_1_), the TG’s probability of *agreeing* and *strongly agreeing* with the statement asserting one's regular engagement in physical activities increased significantly compared to the CG by 9 percentage points (*p* < 0.001; 95% CI: 0.04, 0.14) and 28 percentage points (*p* < 0.001; 95% CI: 0.20, 0.36) respectively; these effects persisted until the end of the trial (Figure 3a). The results confirm hypothesis 1a that, on average, according to participants’ self‐assessment, the fitness‐app prototype contributed to increasing regular physical activity.

Further analysis showed that the treatment effect reflected by the increase in ‘strongly agree’‐responses on being active on a regular basis was driven by two changes in response behaviour. The predicted probability of choosing this response option increased in the TG from 16% to 37% and decreased in the CG from 22% to 14% over the first 4 months (from *t*_0_ to *t*_1_). Response behaviour, however, remained constant in both groups in the following 4 months (from *t*_1_ to *t*_2_).

In addition, ML‐model 2 analyses investigated the effects of the frequency of use (Table S2). Compared to non‐users, the probability of being able to ‘agree strongly’ with being active on a regular basis was 33 percentage points higher (*p* < 0.001; 95% CI: 0.22, 0.43) for *frequent* users of the summary overview of physical activities and 19 percentage points (*p* < 0.001; 95% CI: 0.09, 0.29) for *regular* users. No effect was shown for infrequent users. The results confirmed hypothesis 1b; on average, frequent as well as regular users were more likely to report being physically active on a regular basis than non‐users.

### 4.4 Effects on the frequency of fitness exercises

In terms of the frequency of exercises, we found no significant differences in probabilities between TG and CG at baseline (Table 1, and Figure 3b). After 4 months (*t*_1_), participants in the TG were 45 percentage points (*p* < 0.001; 95% CI: 0.32, 0.57) more likely to state that they exercise ‘more than once a week’ than the CG. Also, TG participants were 34 percentage points (*p* < 0.001; 95% CI: 0.46, 0.22) less likely to report having ‘never’ exercised. After 8 months (*t*_2_), these effects remained (Figure 3b), supporting hypothesis 2a that home care service users with access to the prototyped app reported practicing more often than those without access to the fitness app.

Again, the effect of reporting to ‘exercise more than once a week’ resulted from both an increase in the predicted probability of the TG from 25% to 66% and a decrease in the CG from 21% to 18% after 4 months (*t*_1_). The measured changes in probabilities between TG and CG remained at this level after 8 months (*t*_2_).

The results become more clear‐cut when distinguishing user groups for the apps feature ‘fitness exercises’. The probability of stating they had exercised ‘more than once a week’ was 56 percentage points (*p* < 0.001; 95% CI: 0.43, 0.68) higher for frequent users compared to non‐users at the end of the intervention. Similarly, regular users showed a 30 percentage point (*p* = 0.004; 95% CI: 0.09, 0.50) and infrequent users a 28 percentage point (*p* < 0.001; 95% CI: 0.12, 0.44) higher probability of reporting exercising ‘more than once a week’. This supports hypothesis 2b that frequent and regular (but also infrequent) users of the feature ‘fitness exercises’ report exercising more often than non‐users.

### 4.5 Effects on the frequency of walks

We did not find a significant average treatment effect for the self‐reported frequency of longer walks, either after 4 months (*t*_1_) nor after 8 months *(t*_2_) (Figure 3c). We thus reject hypothesis 3a, as offering access to the fitness‐app prototype did not increase the frequency of walking for 10 min or longer in care‐dependent people.

In contrast to this, the analysis of the usage frequency data revealed a significant effect for frequent and regular users. For frequent users of the activity tracker, the probability of reporting ‘daily’ walks that last at least 10 min was 10 percentage points higher (*p* = 0.021; 95% CI: 0.01, 0.18) compared to non‐users. Regular users’ probability of reporting ‘daily’ walks was 15 percentage points higher (*p* = 0.014; 95% CI: 0.03, 0.27). For infrequent users, no effect could be found. Although frequent and regular users’ probability of reporting daily 10‐min or longer walks exceeded that of non‐users, which supports hypothesis 3b, the effect size did not significantly differ between both user groups.

## 5 DISCUSSION

Digitisation shapes our lives in many areas at an increasing pace, yet less profoundly, in social or long‐term care settings. Despite innovative, low‐cost and scalable technologies to support active and healthy aging being in high demand (Jonkman et al., 2018; Stockwell et al., 2019), older care‐dependent adults have hardly been targeted in eHealth interventions and related research on digital behavioural change techniques (e.g. Elavsky et al., 2019; Muellmann et al., 2018).

Thus, this study is one of the few exploring the effects of a fitness‐app prototype tailored to the needs of older adults using home care services on their self‐assessed levels of physical activity. We found evidence for app‐triggered improvements in self‐reported physical activity. Without the app, care‐dependent people would have kept regular physical activities at a rather low level and (still) the relatively active people in this group would have scaled down on these activities. Having access to the fitness app tailored to the needs of older adults led to positive changes in exercising behaviour that were upheld to the very end of the 8‐month app‐supported fitness intervention. As implemented in the app prototype, for successful interventions, Sullivan and Lachman (2017) highlighted the potential of personalised feedback and (moderate) virtual coaching (e.g. video demonstrations) and Wichmann et al. (2020) additionally pointed to fitting the intervention design to participants’ expectations. A user experience study for this fitness‐app prototype (Trukeschitz & Blüher, 2018b) discovered that the app was well received by home care service users, with one out of every three very likely recommending the fitness app to other older people; however, bugs and misspecifications in the prototype, as well as the lack of adjustable font sizes, left room for technical improvement of the app. We did not find any significant effect on walking behaviour in total, but for the sub‐groups of frequent and regular users of the activity tracker. Accounting for frequency of usage indicated that just having access to a fitness app or an activity tracker does not guarantee sustained behaviour change. To make a difference, individuals have to use these devices on a regular basis.

In comparison to previous mHealth interventions as reviewed by Elavsky et al. (2019), this intervention study stands out with regard to five characteristics: First, the mobile fitness intervention for older adults evaluated in this paper was grounded in behaviour change theory. According to Elavsky et al. (2019), only about one in three previous mHealth interventions (19 out of 52) were theory‐driven.

Second, this intervention study provides evidence on the efficacy of fitness technology for older care‐dependent people, a group that has been largely ignored by previous digital behavioral change intervention research. Our sample comprised 178 participants completing the trial in both arms, which compares to a median sample size of 50 and a mean of 114 for Elavsky et al.’s (2019) pool of 52 mHealth studies. Similarly, Stockwell et al.’s (2019) review of 22 digital behavioural change interventions promoting physical activity in older people reported on a majority of small‐scale studies. Thus, our results are among the minority of adequately powered studies involving care‐dependent people.

Third, this study is among the few with an extensive trial period and evaluates effectiveness after 4 and 8 months. On average, reviews reported that mHealth trials lasted 3.5 months (Elavsky et al., 2019), and the majority of eHealth interventions promoting physical activity in community‐dwelling older adults lasted 4 weeks to 6 months (Jonkman et al., 2018).

Finally, our analysis is among the few studies that also accounted for objectively measured usage of the fitness technologies. According to Eysenbach (2005), one of the fundamental methodological problems in eHealth trials is that a notable proportion of people in the TG will not use the intervention or will use it only sparingly. (Semi‐) automatically generated usage data, however, allowed us to investigate not only the general adoption of the AAL‐system but also whether a certain frequency of use is needed to show effects. The estimation of the treatment effect of the frequency of use revealed that frequent and regular users mainly drove the effect. This further supports the hypothesis that the fitness‐app prototype caused the effects found. As a result, we recommend that future research not only tests effects for the group with access to an AAL‐system, but also effects of the frequency with which the system is actually used.

This study comes with some limitations. The field trial was difficult to set up and, in particular recruiting enough participants turned out to be challenging. Most importantly, we did not achieve full‐randomisation as planned. For an abundance of caution, we accounted for the challenges of implementing the study design by both identifying statistical twins prior to recruitment and adding covariates to the models. As a result, in terms of both group sizes and observable characteristics, as was shown in Table 1 and in more detail (e.g. health, use of ICT) by Trukeschitz and Blüher (2018a), the TG and CG were quite balanced. External validity, however, may be limited to older adults receiving home care services who at least do not reject using new ICTs and are poised to engage in exercise programs. Had more home care service users agreed to participate, a different study design would have been sensible, for example, cross‐over or wait‐list control group designs using a random assignment to the TG and CG. Second, as this evaluation is one of the first focusing on the effects of a fitness‐app prototype on care‐dependent people, it required tailoring the potential outcome indicators to the distinct characteristics of the prototype and the survey tool to the capabilities of the target group. In the end, not many established instruments could be used, which may (currently) limit the comparability of our findings with other studies. Critics of subjective measurement caution that using questionnaires is likely to result in an upward bias in measured physical activity (Kwan et al., 2020; Schrack et al., 2016). However, objective measurement, in particular using accelerometers, has also been critically scrutinised due to lack of precision and comfort (Jonkman et al., 2018; Schrack et al., 2016). Pre‐tests of the tracker used for this intervention revealed inaccurate data, particularly for users with slender wrists (Schneider et al., 2020). Finally, as in the majority of previous studies (Elavsky et al., 2019; Kwan et al., 2020; Sullivan & Lachman, 2017), we did not include a follow‐up measurement at a temporal distance to the completion of the intervention.

Future research should investigate how both user‐oriented development and combining different BCTs in longer‐term interventions using new technologies achieve sustainable behaviour change in older people, particularly in older people with functional limitations. Also, additional evidence on the effects of fitness interventions supported by new technologies is needed to overcome the ‘deficit’ or ‘beyond cure’ perspective on older people with functional limitations and support this group in leading a more active lifestyle.

## CONFLICT OF INTEREST

The authors declare no conflict of interest.

## AUTHOR CONTRIBUTIONS

CS, BT und US were involved in acquiring funding for the CiM project. BT conceived the paper, and supervised data collection and analyses. SE conducted the data analysis. US reviewed the literature. BT and SE drafted parts of the paper. BT, SE, US and CS revised the paper critically for important intellectual content. All authors contributed to the writing and editing of the manuscript for publication, and read and approved the final manuscript. BT finalised the manuscript for submission.

## Supporting information

Tables S1 and S2

## Data Availability

The data that support the findings cannot be made publicly available as data sharing is not covered by the informed consent.
