# Supplementary material for: Exploring the effectiveness of a fitness‐app prototype for home care service users in Austria and Italy
Source: Health Soc Care Community. 2022 Jan 30;30(5):e2884–95. doi: 10.1111/hsc.13733 (PMC9546286; doi:10.1111/hsc.13733)
Supplement: Supplementary file 1 — Tables S1 and S2 [file HSC-30-e2884-s001.docx]

**APPENDIX**

**Table S1** Multilevel ordered logistic regression results, model type 1. *Source*: WU, CiM effectiveness surveys data

|  |  | Physical activities on a regular basis | | Frequency of fitness exercises | | Frequency of walks >10min | |
| --- | --- | --- | --- | --- | --- | --- | --- |
|  | n | 490 | | 491 | | 494 | |
|  |  | Odds Ratio (SE) | 95% CI | Odds Ratio (SE) | 95% CI | Odds Ratio (SE) | 95% CI |
| Treatment Effect | t_0_-t_1_ | 19.68*** (9.00) | 8.02; 48.25 | 35.60*** (23.38) | 9.83; 128.97 | 2.21 (0.97) | 0.93; 5.24 |
|  | t_0_-t_2_ | 20.02*** (9.93) | 7.58; 52.90 | 29.54*** (16.86) | 9.65; 90.41 | 2.54 (1.21) | 0.99; 6.46 |
| Period | t_0_-t_1_ | 0.39** (0.12) | 0.21; 0.72 | 0.66 (0.19) | 0.37; 1.17 | 0.47* (0.15) | 0.25; 0.86 |
|  | t_0_-t_2_ | 0.43** (0.13) | 0.23; 0.79 | 0.61 (0.17) | 0.35; 1.05 | 0.61 (0.21) | 0.31; 1.19 |
| Group (ref. control) | intervention | 0.46 (0.23) | 0.17; 1.24 | 1.39 (0.69) | 0.53; 3.69 | 0.59 (0.31) | 0.21; 1.64 |
| Sex (ref. male) | female | 0.87 (0.41) | 0.34; 2.20 | 1.15 (0.52) | 0.47; 2.80 | 1.07 (0.50) | 0.43; 2.68 |
| Age |  | 1.03 (0.03) | 0.98; 1.09 | 1.01 (0.03) | 0.96; 1.06 | 1.02 (0.30) | 0.97; 1.08 |
| Education (ref. lower secondary) | upper secondary | 0.65 (0.32) | 0.25; 1.72 | 1.72 (0.85) | 0.66; 4.54 | 0.45 (0.24) | 0.16; 1.26 |
|  | post- secondary | 0.85 (0.53) | 0.25; 2.88 | 1.79 (1.08) | 0.56; 5.85 | 0.17** (0.11) | 0.05; 0.59 |
|  | tertiary | 2.22 (1.62) | 0.53; 9.26 | 2.94 (2.09) | 0.72; 11.80 | 0.73 (0.52) | 0.18; 2.99 |
| Country  (ref. Austria) | Italy | 0.14*** (0.07) | 0.05; 0.40 | 0.17*** (0.08) | 0.07; 0.43 | 0.52 (0.25) | 0.20; 1.33 |
| Health |  | 2.13*** (0.36) | 1.54; 2.95 | 1.66* (0.33) | 1.13; 2.45 | 1.46 (0.36) | 0.90; 2.37 |
| (I)ADL-Score |  | 2.42** (0.66) | 1.41; 4.13 | 1.75 (0.58) | 0,91; 3.36 | 4.94*** (1.78) | 2.44; 10.02 |
| Informal carer (ref. ‘no’) | Yes | 0.98 (0.45) | 0.40; 2.41 | 0.76 (0.36) | 0.30; 1.95 | 2.57 (1.41) | 0.88; 7.55 |
| Variance at individual level |  | 4.82 (1.05) | 3.15; 7.40 | 4.07 (1.05) | 2.45; 6.76 | 5.13 (1.15) | 3.31; 7.95 |

Notes: interaction term (group x period), * p<0.05, ** p<0.01, *** p<0.001

**Table S2** Multilevel ordered logistic regression results for regular and frequent user groups, model type 2. *Source*: WU, CiM effectiveness surveys data

|  |  | Physical activities on a regular basis | | Frequency of fitness exercises | | Frequency of walks >10min | |
| --- | --- | --- | --- | --- | --- | --- | --- |
|  | n | 490 | | 491 | | 494 | |
|  |  | Odds Ratio (SE) | 95% CI | Odds Ratio (SE) | 95% CI | Odds Ratio (SE) | 95% CI |
| User group (ref. non-users) | infrequent-users | 3.54 (2.34) | 0.97; 12.90 | 7.89*** (4.50) | 2.57; 24.15 | 2.05 (1.02) | 0.77; 5.45 |
|  | regular-users | 5.85*** (2.49) | 2.53; 13.49 | 8.81** (6.56) | 2.05; 37.89 | 4.11** (2.15) | 1.47; 11.48 |
|  | frequent-users | 16.87*** (7.00) | 7.48; 38.05 | 61.51*** (37.86) | 18.41; 205.52 | 2.56* (0.99) | 1.20; 5.46 |
| Period | t_0_-t_1_ | 0.57* (0.16) | 0.34; 0.97 | 0.90 (0.24) | 0.53; 1.52 | 0.44** (0.12) | 0.26; 0.74 |
|  | t_0_-t_2_ | 0.73 (0.20) | 0.43; 1.25 | 0.92 (0.25) | 0.54; 1.56 | 0.68 (0.18) | 0.40; 1.16 |
| Sex (ref. male) | female | 0.79 (0.35) | 0.33; 1.90 | 1.01 (0.42) | 0.45; 2.28 | 1.05 (0.51) | 0.41; 2.71 |
| Age |  | 1.03 (0.03) | 0.98; 1.08 | 0.99 (0.02) | 0.94; 1.04 | 1.03 (0.03) | 0.97; 1.09 |
| Education  (ref. lower secondary) | upper secondary | 0.51 (0.24) | 0.20; 1.2  7 | 0.95 (0.43) | 0.39; 2.32 | 0.45 (0.24) | 0.16; 1.27 |
|  | post-secondary | 0.80 (0.46) | 0.26; 2.47 | 1.38 (0.73) | 0.49; 3.91 | 0.16** (0.11) | 0.05; 0.59 |
|  | tertiary | 1.59 (1.08) | 0.42; 6.03 | 1.42 (0.92) | 0.40; 5.04 | 0.74 (0.54) | 0.18; 3.10 |
| Country (ref. Austria) | Italy | 0.19** (0.10) | 0.07; 0.51 | 0.26** (0.11) | 0.11; 0.61 | 0.54 (0.26) | 0.20; 1.40 |
| Health |  | 2.00*** (0.32) | 1.46; 2.72 | 1.58* (0.30) | 1.08; 2.31 | 1.42 (0.34) | 0.89; 2.27 |
| IADL-Score |  | 2.35** (0.67) | 1.35; 4.10 | 1.65 (0.54) | 0.86; 3.14 | 4.96*** (1.81) | 2.43; 10.14 |
| Informal carer (ref. ‘no’) | yes | 0.84 (0.34) | 0.38; 1.86 | 0.94 (0.42) | 0.40; 2.24 | 2.21 (1.20) | 0.76; 6.41 |
| Variance at individual level |  | 4.07 (0.92) | 2.62; 6.33 | 3.32 (0.91) | 1.94; 5.68 | 5.45 (1.20) | 3.54; 8.38 |

Notes: * p<0.05, ** p<0.01, *** p<0.001
